# Validation of the Atopic Dermatitis Control Tool (ADCT©) using a longitudinal survey of biologic-treated patients with atopic dermatitis

**DOI:** 10.1186/s12895-019-0095-3

**Published:** 2019-11-06

**Authors:** Eric Simpson, Laurent Eckert, Abhijit Gadkari, Usha G. Mallya, Min Yang, Lauren Nelson, Michelle Brown, Matt Reaney, Puneet Mahajan, Isabelle Guillemin, Mark Boguniewicz, David Pariser

**Affiliations:** 1 Oregon Health and Science University, Portland, OR USA; 2 Sanofi, France, Chilly-Mazarin, Paris, France; 3 Regeneron Pharmaceuticals, Inc, Tarrytown, NY USA; 4 Sanofi, Cambridge, MA USA; 5 Analysis Group, Boston, MA USA; 6 RTI Health Solutions, Research Triangle Park, NC USA; 7 Sanofi, Guildford, UK; 8 Sanofi, Bridgewater, NJ USA; 9 School of Medicine, University of Colorado, Boulder, CO USA; 10 Eastern Virginia Medical School, Norfolk, VA USA

**Keywords:** Atopic dermatitis, tool, control, symptoms, impacts, ADCT©, reliability, validity, responsiveness

## Abstract

**Background:**

The Atopic Dermatitis Control Tool (ADCT©) is a brief patient self-administered instrument designed and validated to assess atopic dermatitis (AD) control; six AD symptoms and impacts are evaluated over the past week, including overall severity of symptoms, days with intense episodes of itching, intensity of bother, problem with sleep, impact on daily activities, and impact on mood or emotions. This study assessed the reliability, validity, and responsiveness of the ADCT in a longitudinal context, and provided thresholds to identify meaningful within-person change.

**Methods:**

Data were from a prospective, longitudinal patient survey study of real-world effectiveness of dupilumab in patients with AD. Eligible patients completed a baseline survey before starting dupilumab and were followed at Months 1, 2, 3, and 6 post-initiation as they became eligible.

**Results:**

Psychometric analyses confirmed internal consistency; Cronbach’s α coefficients were consistently above the threshold of 0.70 across each follow-up; item-to-total correlations were above the threshold of r ≥ 0.50. High correlations between the ADCT and the Dermatology Life Quality Index (DLQI) and skin pain supported construct validity, while known-group validity was shown on Patient Global Assessment of Disease (PGAD) overall well-being subgroups with worse AD-related overall well-being having higher mean ADCT total scores at all time points. The ability of the ADCT to detect change was confirmed; the threshold for meaningful within-person change was estimated to be 5 points. Finally, test–retest reliability was confirmed in subgroups of patients with stable PGAD responses.

**Conclusions:**

Our findings confirm that the ADCT is a valid and reliable tool for assessing AD control.

## Background

Atopic dermatitis (AD) is a systemic, inflammatory skin condition [1, 2] characterized by intense pruritus, eczematous lesions, swelling, and pain [3–6]. With increasing awareness of the substantial patient burden associated with long-term uncontrolled AD [3, 7–13], especially as it relates to sleep disturbance, health-related quality of life, and work or school performance [6, 14–19], assessment of patient self-reported disease control has been deemed crucial for clinical evaluation of AD [15, 20, 21].

Several patient-reported outcome measures (PROMs), including the Peak Pruritus Numerical Rating Scale (Peak Pruritus NRS) [22] for itch, the Patient-Oriented Eczema Measure (POEM) [23] for overall AD symptoms, and the Dermatology Life Quality Index (DLQI) [24] for health-related quality of life (HRQL), are available for use in AD trials; however, these PROMs do not holistically capture the broad concept of disease control. AD control has been described in various ways in the literature, ranging from reduced disease severity or the absence of AD flares, to the impact of AD on patients’ everyday lives and well-being [25–29].

The Atopic Dermatitis Control Tool (ADCT©) is a new PROM designed to assess patient-perceived disease control, meeting this current measurement gap in the management of patients with AD (ADCT v1; https://patient-questionnaires.sanofi.com/questionnaires/adct-atopic-dermatitis-control-communication-tool) (Pariser D, Simpson E, Gadkari A, Bieber T, Margolis D, Brown M, Nelson L, Mahajan P, Reaney M, Guillemin I *et al*: Design, validation and scoring of the Atopic Dermatitis Control Tool (ADCT), unpublished). It is envisaged that the tool will also foster patient–clinician communication regarding disease control. The ADCT is a simple, brief tool that evaluates six symptoms and effects associated with AD over the past week. These include overall severity of symptoms, days with intense episodes of itching, intensity of bother, problem with sleep, impact on daily activities, and impact on mood or emotions. Each of the six ADCT items has a score range from 0 (no problem) to 4 (worst), rating the severity of each concept; the total score ranges from 0 to 24, which is the summation of the responses to all the items. An initial evaluation of the psychometric properties of the instrument in the United States has indicated that the ADCT is valid and reliable for assessing patient-perceived AD control in adults (Pariser D, Simpson E, Gadkari A, Bieber T, Margolis D, Brown M, Nelson L, Mahajan P, Reaney M, Guillemin I *et al*: Design, validation and scoring of the Atopic Dermatitis Control Tool (ADCT), unpublished). In addition, a score of ≥7 points was derived as the threshold to identify patients “not in control”, based on optimal sensitivity/specificity values (Pariser D, Simpson E, Gadkari A, Bieber T, Margolis D, Brown M, Nelson L, Mahajan P, Reaney M, Guillemin I *et al*: Design, validation and scoring of the Atopic Dermatitis Control Tool (ADCT), unpublished). The ADCT© is protected by copyright with all rights reserved to Sanofi and its development partners.

The present study further assessed the reliability, validity, and responsiveness of the ADCT. In addition, it defines a threshold to identify meaningful within-person change. The assessments were conducted on data from the EaRly REal-WorLd PatIent EValuation for DupixEnt in Atopic Dermatitis (RELIEVE-AD) study, a prospective, longitudinal patient survey conducted in the United States that aims to evaluate the early effectiveness of dupilumab in the real-world setting. Dupilumab is approved in the United States and Japan for subcutaneous administration every 2 weeks for the treatment of patients aged ≥12 years with moderate-to-severe AD inadequately controlled with topical prescription therapies or when those therapies are not advisable [30], and in the European Union for use in adults with moderate-to-severe AD who are candidates for systemic therapy [31].

## Methods

### Data source

RELIEVE-AD is an ongoing observational, prospective, longitudinal survey study in adult patients with AD who were enrolled in the Dupixent MyWay™ Patient Support Program and for whom dupilumab had been recently prescribed. Eligible patients completed a baseline survey before starting dupilumab and were followed at Months 1, 2, 3, 6, 9, and 12 post-initiation as they become eligible.

Patient enrollment into the RELIEVE-AD study began in January 2018 and the final data collection is expected to be completed in February 2020. The present study included patients in the RELIEVE-AD study who, on December 6, 2018, had completed the baseline and Months 1, 2, 3, and 6 surveys. Patients were eligible for inclusion in the RELIEVE-AD study if they met the following criteria at the time of the baseline survey:

- aged ≥18 years
- can speak and read English
- be willing to participate in the study and provide informed consent
- have not previously participated in a dupilumab clinical trial
- have not initiated treatment with dupilumab.

The surveys collected data on patient characteristics, including socio-demographics (age, sex, race/ethnicity, marital status, level of education, insurance, employment status, level of income, geographic region), medical history (self-reported age at AD diagnosis, comorbidities), and AD treatment and experience (treatment history prior to dupilumab initiation, concomitant therapy post dupilumab initiation, self-reported adherence to treatment and reasons for discontinuation), and treatment satisfaction.

In addition, PRO data were collected using the Patient Global Assessment of Disease (PGAD), Numerical Rating Scale (NRS) for patient self-reported symptoms (skin pain, burning, and sensitivity) (scores: 0–10; higher scores indicate worse symptom severity), disease control using ADCT (eczema-related symptoms, days with intense itching, overall bothersomeness, sleep problems, daily activities, mood/emotion; total score 0–24; higher scores indicate worse disease control), health-related quality of life (HRQL) using the Dermatology Life Quality Index (DLQI: 0–30, higher scores indicating worse HRQL), and the Work Productivity and Activity Impairment-Atopic Dermatitis questionnaire (WPAI-AD; percentages: 0–100, higher percentages indicate greater impairment) for patients in employment. (Table 1).

**Table 1:** Patient-reported outcome measures used in RELIEVE-AD

Questionnaires	Items (#)	Item content	Response options	Recall period	Score ranging and direction definition
Atopic Dermatitis Control Tool (ADCT)	6	Overall severity of symptoms Days with intense episodes of itching Intensity of bothersomeness Problem with sleep Impact on daily activities Impact on mood or emotions	None, Mild, Moderate, Severe, Very severe Not at all, 1–2 days/nights, 3–4 days/nights, 5–6 days/nights, Every day/Every night Not at all, A little, Moderately, Very/A lot, Extremely	Past week	Item scores ranging from 0 to 4 Total score ranging from 0 (i.e., best disease control) to 24 (i.e., worst disease control) ADCT total score < 7 is considered as AD controlled, whereas ≥7 is considered AD is not controlled
Skin pain NRS	1	Overall skin pain or soreness	NA	Past week	Scores ranging from 0 to 10 0 = No skin pain or soreness 10 = Worst skin pain or soreness
Patient Global Assessment of Disease (PGAD) overall well-being	1	AD-related overall well-being	Excellent, Very good, Good, Fair, Poor	Past week	NA
Dermatology Life Quality Index (DLQI)	10	Itch, pain, stinging symptoms Embarrassment Activities of daily living Everyday life (clothes to wear) Social life Sport activities Work and professional life Relationships Intimate life Treatment on everyday life	Very much, A lot, A little, Not at all	Past week	Total score ranging from 0 (i.e., no effect on patient’s life) to 30 (i.e., largest effect on patient’s life) Bands meaning: 0–1 = no effect at all on patient’s life 2–5 = small effect on patient’s life 6–10 = moderate effect on patient’s life 11–20 = very large effect on patient’s life 21–30 = extremely large effect on patient’s life
Work Productivity and Activity Impairment - Atopic Dermatitis (WPAI-AD)	6	Absenteeism Presenteeism Total work impairment Total activity impairment	Multiple formats: Dichotomous Yes/No; NRS; number of hours	Past 7 days	Total score ranging from 0% impairment to 100% impairment

*AD* Atopic dermatitis, *NA* Non-applicable, *NRS* Numerical rating scale

Analyses in this study were conducted using PRO data from multiple survey timepoints to ensure the robustness of the findings.

### Statistical methods

All data analyses were conducted using SAS 9.4 (SAS Institute Inc., Cary, NC, USA).

#### Reliability

Assessments for reliability included internal consistency reliability and test–retest reliability. Internal consistency was assessed using Cronbach’s alpha (α ≥0.7) [32]. ADCT item-to-total correlations were estimated at baseline and Months 1, 2, 3, and 6 using the Pearson correlation coefficient (PCC ≥0.5) [33]. Test–retest reliability was evaluated based on the intra-class correlation (ICC) coefficient of the ADCT total score among patients with unchanged PGAD scores across month pairs (between Months 1 and 2, Months 2 and 3, and Months 3 and 6). An ICC ≥0.70 was expected for confirming test–retest reliability [34].

#### Construct validity

Convergent validity of the ADCT was assessed by computing Spearman’s rank-order correlation between the ADCT total score and the DLQI (total and item-level scores), skin pain, PGAD overall well-being, WPAI total work impairment (WPAI-TWI), and WPAI total activity impairment (WPAI-TAI) at baseline and Months 1, 2, 3, and 6. Given that the skin pain NRS directly measures AD-related symptoms and the DLQI includes questions on both symptoms and impacts due to skin problems, correlations between the ADCT and skin pain and DLQI were expected to be higher than the correlations between the ADCT and other measures, such as PGAD, WPAI-TWI, and WPAI-TAI. Cohen’s recommended guidelines for determining small, moderate, or large effects (0.1 to < 0.3, 0.3 to < 0.5, and ≥ 0.5, respectively) were applied, and a large effect (r ≥ 0.5) was used in this study as evidence of convergent validity [35]. Divergent validity, established previously for the ADCT, was not assessed here owing to the lack of appropriate measures for use from the current study.

#### Known-groups validity

To confirm known-groups validity, mean ADCT total scores were compared across adjacent subgroups of patients based on PGAD responses (Excellent, Very good, Good, Fair, Poor) and categories of DLQI responses: no effect on patient life (score range: 0–1), a small effect (2–5), a moderate effect (6–10), a very large effect (11–20), or an extremely large effect (21–30) [36] (Table 1). Patients in a worse PGAD or DLQI band subgroup were expected to display poorer AD control (i.e., higher mean ADCT total scores, indicating more severe symptoms/greater impact) than patients in a better PGAD or DLQI band subgroup. If the homogeneity of variance across the subgroups was rejected (*p* < 0.05) based on a Levene’s test of equality of variance, a Mann–Whitney U test was used to compare the mean ADCT total scores between the subgroups; otherwise, *t*-tests were applied. Cohen’s d was calculated for the standardized differences in mean ADCT total scores between subgroups and was corrected for small sample sizes when the total sample size in the two groups was below 50 [37].

#### Ability to detect change (responsiveness)

Responsiveness was evaluated using correlations between the change from baseline (to Months 1, 2, 3, and 6) in ADCT total score and the change from baseline in DLQI total score (Pearson product-moment). The same analysis was conducted using (Spearman’s rank-order correlation) (r ≥ 0.5) for DLQI bands and PGAD scores [34].

#### Interpretation of change

Anchor-based and distribution-based methods were used to establish a threshold characterizing meaningful within-person change in the ADCT total score.

Prior to applying the anchor-based method, the correlation coefficient between the change in the ADCT total score and the potential anchor measure was reviewed for the magnitude of association; in this study, a large effect (i.e., correlation at least 0.5) was required [38]. Once established as appropriate, univariate regression analyses accounting for repeated measures were conducted; changes in ADCT total scores from baseline was the dependent variable and changes in the anchor measure from baseline was the independent variable. The change in PGAD and change in DLQI were considered as potential anchor measures and the following anchors were selected a priori: a 1-level improvement in the PGAD; a 4-point improvement on the DLQI total score [39]; or a 1-level improvement in the DLQI band. Patients who were not likely to change were excluded: e.g., reporting PGAD = “excellent” or DLQI = “no effect” (i.e., total score of 0 or 1) at baseline. Additional analysis was conducted using the subset of patients whose AD was considered not controlled at baseline based on the ADCT total score (i.e., score > 7; Table 1), as established in previous research (Pariser D, Simpson E, Gadkari A, Bieber T, Margolis D, Brown M, Nelson L, Mahajan P, Reaney M, Guillemin I *et al*: Design, validation and scoring of the Atopic Dermatitis Control Tool (ADCT), unpublished).

For the distribution-based approach, the half standard deviation (SD) method of the baseline ADCT scores, one-third SD, one unit of standard error of measurement (SEM), and two units of SEMs were examined. Final recommendations for thresholds characterizing meaningful within-person change and considered as a clinical important responder were made considering the anchor- and distribution-based results.

## Results

### Patient population

The interim dataset from RELIEVE-AD, as of December 6, 2018, included 1010 patients who completed the baseline survey, 538 patients at Month 1, 458 patients at Month 2, 372 patients at Month 3, and 206 patients at Month 6. Patients who were eligible to receive the survey at each timepoint varied based on time elapsed since they initiated dupilumab. Accounting for the number of surveys sent out at each timepoint, the response rate ranged from between 89.8% in Month 1 to 74.4% in Month 6. The smaller sample sizes in the later follow-ups were attributable to many patients not due for survey completion at the time of this interim data cut. Overall, patient characteristics were comparable between patients at baseline and those who had completed the follow-up surveys.

At baseline, the mean age of the patients was 47 years, and the mean age at AD diagnosis was 28 years. More than half of the population (62%) were female and the majority (74%) were White. Most patients (96%) reported experiencing flares over the previous 4 weeks at baseline. The mean skin pain NRS score was 5.9 and the mean DLQI total score was 13.4; no patients reported a DLQI score of 0 or 1. Very few patients (3.4%) reported levels of ‘excellent’ or ‘very good’ on the PGAD. The mean WPAI-TAI and WPAI-TWI were 45.8 and 40.8%, respectively. The mean ADCT total score was 15.9 at baseline.

### Reliability

Cronbach’s α coefficients of the ADCT total score were 0.90 at baseline, 0.93 at Month 1, 0.94 at Month 2, 0.9 at Month 3, and 0.95 at Month 6. Item-to-total correlations ranged from 0.68 to 0.81 at baseline to 0.79 to 0.88 at Month 6 (Table 2). ICCs computed using subgroups of patients with stable PGAD responses were 0.82 for assessments between Months 1 and 2 (*n* = 219), 0.78 between Months 2 and 3 (*n* = 189), and 0.79 between Months 3 and 6 (*n* = 107) (Table 3).

**Table 2:** Cronbach’s α for internal consistency reliability of ADCT

	Baseline (*n* = 1010)	Month 1 (*n* = 538)	Month 2 (*n* = 458)	Month 3 (*n* = 372)	Month 6 (*n* = 206)
Overall internal consistency	0.90	0.93	0.94	0.94	0.95
Item-to-total correlation					
Overall severity of AD symptoms	0.74	0.79	0.81	0.81	0.84
Days with intense episodes of itching	0.68	0.76	0.79	0.81	0.79
Intensity of bothersomeness	0.81	0.85	0.89	0.89	0.88
Problem with sleep	0.68	0.75	0.73	0.78	0.83
Impact on daily activities	0.76	0.82	0.82	0.84	0.82
Impact on mood or emotions	0.71	0.79	0.82	0.84	0.86

*AD* Atopic dermatitis, *ADCT* Atopic Dermatitis Control Tool

**Table 3:** Test–retest reliability of ADCT anchored by no change in the Patient Global Assessment of Disease

	*N*	ICC (95% CI)
ADCT total score at Months 1 and 2	219	0.82 (0.77, 0.86)
ADCT total score at Months 2 and 3	189	0.78 (0.71, 0.83)
ADCT total score at Months 3 and 6	107	0.79 (0.71, 0.85)

*ADCT* Atopic Dermatitis Control Tool, *CI* Confidence interval, *ICC* Intra-class correlation coefficient

### Construct validity

The highest correlations were observed between the ADCT total score and skin pain NRS (from 0.74 to 0.83) and the DLQI total score in the follow-up surveys (from 0.80 to 0.85), supporting construct validity (Table 4). Spearman’s rank-order correlations between the ADCT total score and individual DLQI items ranged between 0.37 (issues at work or studying) and 0.75 (degree of itchiness, soreness, pain or sting) at baseline to 0.12 (issues at work or studying) and 0.75 (degree of itchiness, soreness, pain or sting) at Month 6. Other item correlations ranged between 0.4 and 0.6 regardless of the timepoint.

**Table 4:** Construct validity with Spearman’s rank-order correlations between ADCT total score and other patient-reported outcome measures in RELIEVE-AD

	Baseline	Month 1	Month 2	Month 3	Month 6
DLQI total score	0.543***	0.803***	0.828***	0.846***	0.812***
PGAD	0.489***	0.734***	0.762***	0.758***	0.705***
Skin pain	0.741***	0.831***	0.807***	0.793***	0.806***
WPAI-TAI	0.645***	0.677***	0.750***	0.772***	0.706***
WPAI-TWI	0.605***	0.644***	0.686***	0.721***	0.725***

***p < 0.001

*ADCT* Atopic Dermatitis Control Tool, *DLQI* Dermatology Life Quality Index, *PGAD* Patient Global Assessment of Disease, *WPAI-TAI* Work Productivity and Activity Impairment - Total Activity Impairment, *WPAI-TWI* Work Productivity and Activity Impairment - Total Work Impairment

### Known-group validity

Known-group analyses indicated that PGAD subgroups with worse AD-related overall well-being had higher mean ADCT total scores (poor AD control) at all timepoints (Table 5). The differences in mean ADCT total score between the adjacent groups were statistically significant (*p* < 0.01), except between ‘excellent’ and ‘very good’ at baseline and between ‘fair’ and ‘poor’ at Month 6, likely due to small sample sizes. Similarly, patients in the groups of DLQI bands with greater effect on life were associated with higher mean ADCT total scores (poor AD control) (Table 5). All differences in mean ADCT total score between the adjacent bands were statistically significant (*p* < 0.05) except between the small effect and the moderate effect bands at baseline, and between the very large effect and the extremely large effect bands at Month 6. The Cohen’s d effect size showed large effect across all adjacent categories except between ‘excellent’ and ‘very good’ at baseline, between the small effect and the moderate effect bands at baseline, and between the very large effect and the extremely large effect bands at Month 6.

**Table 5:** Comparisons of mean differences in ADCT total score by PGAD and DLQI known groups

Assessment period	Subgroups	*N*	ADCT total score, mean (SD)	Mean ADCT total score differences (95% CI)		*p*-value	Cohen’s d
Patient Global Assessment of Disease (PGAD)							
Baseline	Excellent	3	8.67 (7.64)	–	–	–	–
	Very good	31	9.90 (5.88)	Excellent vs very good	−1.24 (−8.64, 6.16)	0.736	−0.17^a^
	Good	240	12.85 (5.44)	Very good vs good	−2.95 (−5.01, − 0.89)	0.005	−0.52
	Fair	496	15.79 (4.64)	Good vs fair	−2.94 (−3.70, −2.18)	< 0.001	−0.58
	Poor	240	19.83 (3.97)	Fair vs poor	−4.03 (−4.72, −3.35)	< 0.001	−0.93
Month 1	Excellent	71	1.63 (2.02)	–	–	–	–
	Very good	177	3.85 (2.72)	Excellent vs very good	−2.22 (−2.92, −1.52)	< 0.001	−0.93
	Good	175	7.27 (4.00)	Very good vs good	−3.42 (−4.14, −2.70)	< 0.001	−1.00
	Fair	101	11.40 (4.63)	Good vs fair	−4.12 (−5.17, −3.08)	< 0.001	−0.95
	Poor	14	16.29 (4.05)	Fair vs poor	−4.89 (−7.47, −2.31)	< 0.001	−1.12
Month 3	Excellent	89	1.46 (2.09)	–	–	–	–
	Very good	126	3.64 (2.53)	Excellent vs very good	−2.18 (−2.83, −1.54)	< 0.001	−0.94
	Good	98	7.58 (4.59)	Very good vs good	−3.94 (−4.89, −2.99)	< 0.001	−1.06
	Fair	52	11.27 (4.63)	Good vs fair	−3.69 (−5.25, −2.13)	< 0.001	−0.80
	Poor	7	19.57 (2.37)	Fair vs poor	−8.30 (−11.89, −4.72)	< 0.001	−2.26
Month 6	Excellent	57	1.58 (1.93)	–	–	–	–
	Very good	71	3.23 (2.13)	Excellent vs very good	−1.65 (−2.36, −0.93)	< 0.001	−0.81
	Good	57	6.81 (3.99)	Very good vs good	−3.58 (−4.67, −2.49)	< 0.001	−1.12
	Fair	16	12.75 (5.41)	Good vs fair	−5.94 (−8.39, −3.50)	< 0.001	−1.25
	Poor	5	17.40 (3.36)	Fair vs poor	−4.65 (−10.06, 0.76)	0.088	−0.94^a^
Dermatology Life Quality Index (DLQI) bands							
Baseline	No effect	0	–	–	–	–	–
	Small	21	13.67 (4.86)	–	–	–	–
	Moderate	351	12.74 (5.26)	Small vs moderate	0.92 (−1.39, 3.24)	0.433	0.18
	Very large	511	16.85 (4.66)	Moderate vs very large	−4.11 (−4.78, −3.44)	< 0.001	−0.83
	Extremely large	127	20.79 (3.48)	Very large vs extremely large	−3.94 (−4.80, −3.07)	< 0.001	−0.96
Month 1	No effect	137	2.05 (1.82)				
	Small	214	5.25 (3.08)	No vs small	−3.20 (−3.77, −2.63)	< 0.001	−1.26
	Moderate	107	8.66 (3.75)	Small vs moderate	−3.41 (−4.18, −2.64)	< 0.001	−0.99
	Very large	67	13.43 (4.39)	Moderate vs very large	−4.77 (−6.00, −3.54)	< 0.001	−1.17
	Extremely large	13	16.77 (3.49)	Very large vs extremely large	−3.34 (−5.91, −0.76)	0.012	−0.84
Month 3	No effect	136	1.75 (1.78)	–	–	–	–
	Small	131	4.83 (2.83)	No vs small	−3.08 (−3.65, −2.52)	< 0.001	−1.31
	Moderate	65	8.71 (3.78)	Small vs moderate	−3.88 (−4.82, −2.93)	< 0.001	−1.16
	Very large	30	14.00 (4.18)	Moderate vs very large	−5.29 (−7.00, −3.58)	< 0.001	−1.33
	Extremely large	10	19.80 (2.35)	Very large vs extremely large	−5.80 (−8.63, −2.97)	< 0.001	−1.64^a^
Month 6	No effect	95	1.91 (1.79)	–	–	–	–
	Small	64	4.61 (3.24)	No vs small	−2.70 (−3.50, −1.91)	< 0.001	−1.03
	Moderate	27	8.74 (3.39)	Small vs moderate	−4.13 (−5.63, −2.63)	< 0.001	−1.25
	Very large	18	14.06 (4.52)	Moderate vs very large	−5.31 (−7.69, −2.94)	< 0.001	−1.28^a^
	Extremely large	2	16.50 (7.78)	Very large vs extremely large	−2.44 (−9.89, 5.01)	0.499	−0.35^a^

^a^Cohen’s d was corrected for small sample sizes. The correction factor was (N–3)/(N–2.25) x sqrt (1–2/N)

*ADCT* Atopic Dermatitis Control Tool, *CI* Confidence interval, *SD* Standard deviation

### Ability to detect change (responsiveness)

Correlational analyses confirmed the ADCT’s ability to detect change (responsiveness; Table 6). Specifically, Spearman’s rank-order correlation between change in ADCT total score and change in PGAD from baseline ranged from 0.54 in Month 3 to 0.60 in Month 6. Spearman’s rank-order correlation between change in ADCT total score and change in DLQI bands from baseline ranged from 0.47 in Month 1 to 0.51 in Month 3. Pearson product-moment correlation between change in ADCT total score and change in DLQI total score from baseline ranged from 0.55 in Month 1 to 0.61 in Month 3. All correlation coefficients were statistically significant (*p* < 0.001).

**Table 6:** Responsiveness of ADCT according to change in Patient Global Assessment of Disease (PGAD) and Dermatology Life Quality Index (DLQI)

	Correlation coefficient
Change in ADCT total score vs. change in PGAD	Spearman (95% CI)
Month 1 vs. Baseline, *n* = 538	0.56*** (0.50, 0.62)
Month 2 vs. Baseline, *n* = 458	0.58*** (0.51, 0.64)
Month 3 vs. Baseline, *n* = 372	0.54*** (0.46, 0.61)
Month 6 vs. Baseline, *n* = 206	0.60*** (0.50, 0.68)
Change in ADCT total score vs. change in DLQI bands	Spearman (95% CI)
Month 1 vs. Baseline, *n* = 538	0.47*** (0.40, 0.54)
Month 2 vs. Baseline, *n* = 458	0.48*** (0.41, 0.55)
Month 3 vs. Baseline, *n* = 372	0.51*** (0.43, 0.58)
Month 6 vs. Baseline, *n* = 206	0.50*** (0.39, 0.59)
Change in ADCT total score vs. change in DLQI total score	Pearson (95% CI)
Month 1 vs. Baseline, *n* = 538	0.55*** (0.49, 0.61)
Month 2 vs. Baseline, *n* = 458	0.57*** (0.50, 0.63)
Month 3 vs. Baseline, *n* = 372	0.61*** (0.54, 0.67)
Month 6 vs. Baseline, *n* = 206	0.60*** (0.51, 0.68)

***p < 0.001

*ADCT* Atopic Dermatitis Control Tool, *CI* confidence interval

### Interpretation of change

Changes in PGAD, DLQI bands, and DLQI total score correlated well with change in ADCT total score (r > 0.50); therefore, PGAD and DLQI were determined to be appropriate anchors. Through the anchor-based approach, 1-level improvement in PGAD or in DLQI bands, or a 4-point reduction in DLQI total score, was associated with a reduction in ADCT total score of 5.30, 5.20, or 3.90, respectively, among the overall sample, and 5.43, 5.42, or 4.03, respectively, among patients with uncontrolled AD symptoms at baseline (Table 7).

**Table 7:** Average reduction in ADCT total score by improvement in Patient Global Assessment of Disease (PGAD) and Dermatology Life Quality Index (DLQI)

	Overall sample		Uncontrolled patients (ADCT ≥7 at baseline)	
	*N*	Mean reduction	*N*	Mean reduction
Reduction in ADCT total score per 1-level improvement in PGAD^a^	1570	5.30*** (5.14, 5.46)	1479	5.43*** (5.27, 5.59)
Reduction in ADCT total score per 1-level improvement in DLQI bands^b^	1574	5.20*** (5.05, 5.35)	1483	5.42*** (5.27, 5.58)
Reduction in ADCT total score per 4-point improvement in DLQI total score^b^	1574	3.90*** (3.80, 4.00)	1483	4.03*** (3.92, 4.12)

***p < 0.001; ^a^Patients who reported excellent PGAD at baseline were excluded from this analysis due to lack of variability; ^b^No patients had 0 or 1 DLQI scores at baseline and therefore no patients were excluded from this analysis

*ADCT* Atopic Dermatitis Control Tool

Using the distribution-based approach, the half SD and one-third SD of ADCT total score at baseline were 2.72 and 1.81, respectively. The SEM of ADCT total score at baseline was 1.71 when using the overall Cronbach’s α at baseline as reliability measure and 2.32 when using the ICC between Month 1 and Month 2 as reliability measure. Consequently, 2 units of SEM were 3.42 and 4.64, respectively.

## Discussion

Practice guidelines recommend that during a clinical evaluation, clinicians inquire about a patient’s itch, sleep, and impact on daily activity due to their AD [15]. However, no single PROM is currently available to holistically evaluate these concepts within a single tool. The ADCT was previously developed and validated to assess AD control, with the standards recommended in the PRO Guidance by the US Food and Drug Administration [40]. Not only does this PROM evaluate AD control in a comprehensive and standardized approach, but, at the same time, it can easily be completed at home or during a clinical encounter given its brevity and ability to be self-administered via paper, online, or handheld device. The ADCT is brief, straightforward, and easily scored and interpreted, providing an immediate metric to patient self-measure of their disease control, and is thus very well adapted to clinical practice. It is anticipated that the ADCT will also facilitate meaningful patient–clinician dialogue about disease control, enhancing clinical monitoring and informing treatment decisions.

The measurement properties of the ADCT based on initial evaluations have been previously published (Pariser D, Simpson E, Gadkari A, Bieber T, Margolis D, Brown M, Nelson L, Mahajan P, Reaney M, Guillemin I *et al*: Design, validation and scoring of the Atopic Dermatitis Control Tool (ADCT), unpublished); in the present study, we have further evaluated this novel PROM based on data from RELIEVE-AD, a real-world, prospective, longitudinal patient survey. Cross-sectional properties previously defined were confirmed within this longitudinal context. Internal consistency was met, and moreover, ICCs computed using subgroups of patients with stable PGAD responses supported the test–retest reliability of the ADCT total score. High correlations between the ADCT and DLQI and skin pain NRS supported convergent validity, while known-groups validity was shown on PGAD subgroups with worse AD-related overall wellbeing and in the groups of DLQI bands with greater effect on life having higher mean ADCT total scores at all timepoints. Separately, versus the DLQI, the ADCT was not strongly correlated with the item “impact at work or studying” but was strongly correlated with “impact on social or leisure activities” at each of the timepoints. Analyses revealed a very strong correlation with the item “degree of itchiness, soreness, pain or sting”. The total scores were as well strongly correlated. From these findings, it appears that self-perception of disease control is not strongly associated with AD impact at work or studying but it is very strongly with HRQL.

The ability of the ADCT to detect change was confirmed for use in real-world settings; the threshold for meaningful within-person change was estimated to be 5 points. Establishing this threshold allows the clinician to assess clinically meaningful changes in AD control over time based on repeated administration of the ADCT. As previously established, a total score of ≥7 on the ADCT allows a cross-sectional assessment of lack of AD control at a given timepoint (Pariser D, Simpson E, Gadkari A, Bieber T, Margolis D, Brown M, Nelson L, Mahajan P, Reaney M, Guillemin I *et al*: Design, validation and scoring of the Atopic Dermatitis Control Tool (ADCT), unpublished). The meaningful within-person change threshold of 5 points compliments this by allowing a longitudinal assessment of improvement in disease control of a patient over time. Finally, good stability of the ADCT in providing reliable data over time was observed through test–retest scores against subgroups of patients with stable PGAD responses.

In consideration of our positive findings on the validity and reliability of the ADCT, a few study limitations are to be noted. First, participant diagnosis of AD relied only on self-report (i.e., not confirmed by a clinician). However, all included patients were prescribed dupilumab, which was approved only for AD when patients were enrolled in the study. Regarding the sample size, the RELIEVE-AD study is ongoing, and the full dataset is still maturing; therefore, there was a reduction of patient numbers across follow-up periods that was mainly due to the number of patients who were eligible for survey completion at those timepoints by the December 6, 2018 data cut.

While our results confirmed the psychometric properties of ADCT using data from an ongoing observational, prospective, longitudinal patient survey study, additional studies that are currently ongoing will also be able to confirm the present findings and potentially address some of the limitations. For example, one study is currently ongoing including patients and physicians to determine how ADCT compares with clinical scales (e.g., Eczema Area and Severity Index, physician assessment of control). We expect that data from such a study will also be able to confirm our findings within a population of patients with physician-confirmed diagnosis of AD.

Despite the limitations, the real-world survey data used in the present study have added further evidence to initial evaluations that the ADCT is a valid and reliable tool for assessing patient-perceived AD control and may provide a useful patient–clinician communication tool on disease control in clinical and non-clinical settings.

## Conclusion

Our findings confirm that the ADCT is a valid and reliable tool for assessing AD control in real-world, longitudinal settings. In addition, ongoing studies will be able to further evaluate the present findings and potentially address some of the limitations noted.

## Data Availability

The datasets generated and/or analysed during the current study are not publicly available as the RELIEVE-AD study is ongoing with continued data collection, but are available from the corresponding author on reasonable request.

## Abbreviations

AD: Atopic dermatitis
ADCT: Atopic Dermatitis Control Tool
CI: Confidence interval
DLQI: Dermatology Life Quality Index
HRQL: Health-related quality of life
ICC: Intra-class correlation
NRS: Numerical Rating Scale
PCC: Pearson correlation coefficient
PGAD: Patient Global Assessment of Disease
POEM: Patient-Oriented Eczema Measure
PROM: Patient-reported outcome measure
RELIEVE-AD: EaRly REal-WorLd PatIent EValuation for DupixEnt in Atopic Dermatitis
SD: Standard deviation
SEM: Standard error of measurement
WPAI-AD: Work Productivity and Activity Impairment - Atopic Dermatitis
WPAI-TAI: WPAI total activity impairment
WPAI-TWI: WPAI total work impairment
